# CONTRIBUTING FACTORS TO POST-RETIREMENT EMPLOYMENT AND LIFE SATISFACTION

**DOI:** 10.1093/geroni/igae098.4327

**Published:** 2024-12-31

**Authors:** Zhong Wang

**Affiliations:** Yeshiva University, New York, New York, United States

## Abstract

This study explores the factors influencing post-retirement employment and its impact on retirement satisfaction, aiming to provide insights to improve older adults’ productivity and well-being, thereby enhancing their life satisfaction and contributing to the cohesion of families, communities, and society at large. Utilizing secondary data from the RAND HRS Longitudinal File 2020, this quantitative research examines a sample of 1,561 individuals born in 1948 and 1949. The longitudinal data tracks changes in retirement trajectories and self-reported well-being over time. Logistic regression analysis revealed several key findings: African American and Hispanic individuals were less likely to intend to work beyond age 65 but more likely to engage in post-retirement employment. Higher household wealth correlated with a decreased intention to work past 65 but an increased likelihood of continued labor market participation. The health and employment status of a spouse significantly impacted older adults’ work intentions, retirement decisions, and satisfaction. Additionally, there were significant reciprocal relationships between respondents’ work plans and their retirement decisions. The findings highlight the need to address the diverse job-related needs of older adults in gerontological social work practice. Advocating for employment as a human right, the study highlights the importance of age-friendly policies, such as flexible work arrangements and phased retirement options. Social work practice can enhance opportunities for older adults through financial literacy programs, job training, and career counseling, particularly in underserved communities. Additionally, acknowledging the correlation between family dynamics and retirement experiences, gerontological social work can better support older adults in navigating their retirement transitions.

